# A new subspecies of *Avitta fasciosa* Moore, 1882 (Lepidoptera, Erebidae) from Korea

**DOI:** 10.3897/BDJ.13.e176317

**Published:** 2025-12-16

**Authors:** Hee Han, Sora Kim

**Affiliations:** 1 Jeonbuk National University, Jeonju, Republic of Korea Jeonbuk National University Jeonju Republic of Korea

**Keywords:** biogeography, Erebinae, Heuksando, Korean Peninsula, migration

## Abstract

**Background:**

The genus *Avitta* Walker is a quite well-known taxon due to the commitment of previous cladistic research, which was considered in terms of biogeography and included a number species (13 valid species and two undescribed female-based species). Given that there are 18 named species, Holloway's work is significant not only from a biogeographic perspective, but also from a taxonomic and phylogenetic standpoint.

**New information:**

In this study, we recorded a new subspecies, *Avitta fasciosa koreana* **ssp. nov.**, found on a southern island of Korea, with illustrations and genital features. This is not only just a recording, but also a demonstration that the moth species’ samples, which are generally considered tropical species found in East Asia, can be a separate population or species, but not just the results of migration.

## Introduction

In a pioneering biogeographical analysis of the genus *Avitta* Walker (1858) from Asia and Melanesia on the basis of cladistic analysis, Holloway (1984) described four new species and one new subspecies. In his strictly reviewed research for *Avitta*, he found that there are distinct differences of genital features between *A. fasciosa* Moore (1882) from the northeast Himalaya and Borneo (he named each as the typical mainland Asian form and the Sundaland form). Two bioregions are quite distant, but he considered those two samples just “forms or variations”, not taxonomically separated taxa. Considering there are currently 18 described species (Savela 2025), Holloway’s work has not only given a meaning to biogeography, but is also of taxonomic and phylogenetic importance. About 20 years later, Holloway (2005) elevated the Borneo form to a new subspecies and named it *A. f. gracilis* Holloway. He presented its slender costal processes and valva, which are narrow and lacks spurs, as the new subspecies’ autapomorphy. Given that both collecting sites of the holotype specimen of *A. fasciosa* Moore (Khasia Hills, India) and the sample of *A. fasciosa* that Holloway examined as a typical mainland Asian form (Himalaya) are from mainland Asia, it appears that he had maybe thought *A. f. gracilis* could be sufficiently considered as a separate taxon not only morphologically, but also biogeographically. However, we could not find the precise reason why this had just been treated as a new subspecies, not a new species. After that, there is no further taxonomic study handling the genus *Avitta*.

In the present study, we reported a new subspecies, *Avitta fasciosa koreana* **ssp. nov.**, from Korea. The Island of Heuksando is a distantly isolated area from the Korean Peninsula mainland and it is considered as one of the destinations of migrated species by periodic wind or typhoons (Choi et al. 2024, Choi et al. 2025). The new subspecies of this paper just could be treated as one of the results of migration, but we excavated it from the bunch of moths and here we present a new possibility. There are many unofficial records of this species spread throughout both East Asia and Southeast Asia (e.g. iNaturalist), but there is a lack of morphological review of this species. We provide illustrations, a diagnosis and a description of the new subspecies and imply not only the possibility of separating this species in future studies, but also the necessity of further integrative studies including molecular data (e.g. barcoding genes, SNP datasets, etc.) and biogeographic perspectives.

## Materials and methods

The materials utilised in this study, including dried specimens and slide vouchers, have been deposited at the following institute: Laboratory of Insect Phylogenetics & Evolution, Jeonbuk National University (IPE JBNU), Republic of Korea.

The procedure for genitalia preparation for slide vouchers was conducted in accordance with the methodology proposed by Kononenko and Han (2007). All dried specimens and slide vouchers of genitalia were examined under a Canon EOS 6D DSLR camera and an EF 100 mm F 2.8 Macro Lens (Canon Inc., Japan) and images were taken with the same equipment. Multi-stacked images were produced using Helicon Focus and Helicon Remote software (HeliconSoft, Ukraine). The final illustrations were processed using Adobe Photoshop Lightroom Classic and Adobe Photoshop 2024 (Adobe Systems Inc., USA).

## Taxon treatments

### Avitta

Walker, 1858

2C8C10AC-7C45-50FA-B5EA-8FE45E7F4CF6

Avitta Walker 1858, type species: *Avitta subsignans Walker 1858*.
Oroba Walker (1863).

#### Diagnosis

This genus can be defined by the following characteristics: in habitus, fore-wings with several parallel fasciae which are diffused and irregular; reniform bilobed with paler centre; in male genitalia, costa of valva with spur at base; in female genitalia, corpus bursae spherical, finely scobinated entirely and those thicker in patches near base; bearing sclerotised plate between ovipositor lobes (Holloway 1984).

#### Notes

In Korea, only *Avitta puncta* Wileman was reported by Choi (2010). He treated the genus *Asta* Walker as one of the synonyms of *Avitta*. It seems that he followed the classification of Poole (1989). However, after that, Holloway (2005) treated it as the genus Asta, based on the characteristics of habitus and male genitalia. Therefore, we re-examined it and suggest that the position of *A. puncta* should be treated as *Asta*. According to Holloway (2005), he transferred *Avitta puncta* to *Asta*, based on the features of its fore-wing facies and male genitalia. He maintained this taxonomic status in his later checklist of the moths of Borneo (Holloway 2011). Consequently, *Avitta fasciosa koreana* is the only member of *Avitta* in Korea.

### Avitta fasciosa koreana n.

DCF2B8FC-CD7B-54DB-B805-7DD093E0345E

C8EAB087-484B-4D07-8E28-DB917687B24B

#### Materials

- **Type status:** Holotype. **Occurrence:** recordedBy: H. Han et al.; sex: 1 male; occurrenceID: 902F6C56-B702-5D48-A4B3-044BA9C4940D; **Taxon:** kingdom: Animalia; phylum: Arthropoda; class: Insecta; order: Lepidoptera; family: Erebidae; genus: Avitta; specificEpithet: fasciosa; infraspecificEpithet: koreana; taxonRank: subspecies; **Location:** country: South Korea; stateProvince: Jeollanam-do; locality: Sinan-gun, Heuksan-myeon, Sa-ri; decimalLatitude: 34.649250; decimalLongitude: 125.420389; **Event:** eventDate: 03-09-2024
- **Type status:** Paratype. **Occurrence:** recordedBy: H. Han et al.; sex: 1 female; occurrenceID: 65CB272D-AA98-5CBB-9E90-2D388DACE232; **Taxon:** kingdom: Animalia; phylum: Arthropoda; class: Insecta; order: Lepidoptera; family: Erebidae; genus: Avitta; specificEpithet: fasciosa; infraspecificEpithet: koreana; taxonRank: subspecies; **Location:** country: South Korea; stateProvince: Jeollanam-do; locality: Sinan-gun, Heuksan-myeon, Ye-ri; decimalLatitude: 34.665194; decimalLongitude: 125.414694; **Event:** eventDate: 02-09-2024

#### Description

**Figure 1.**
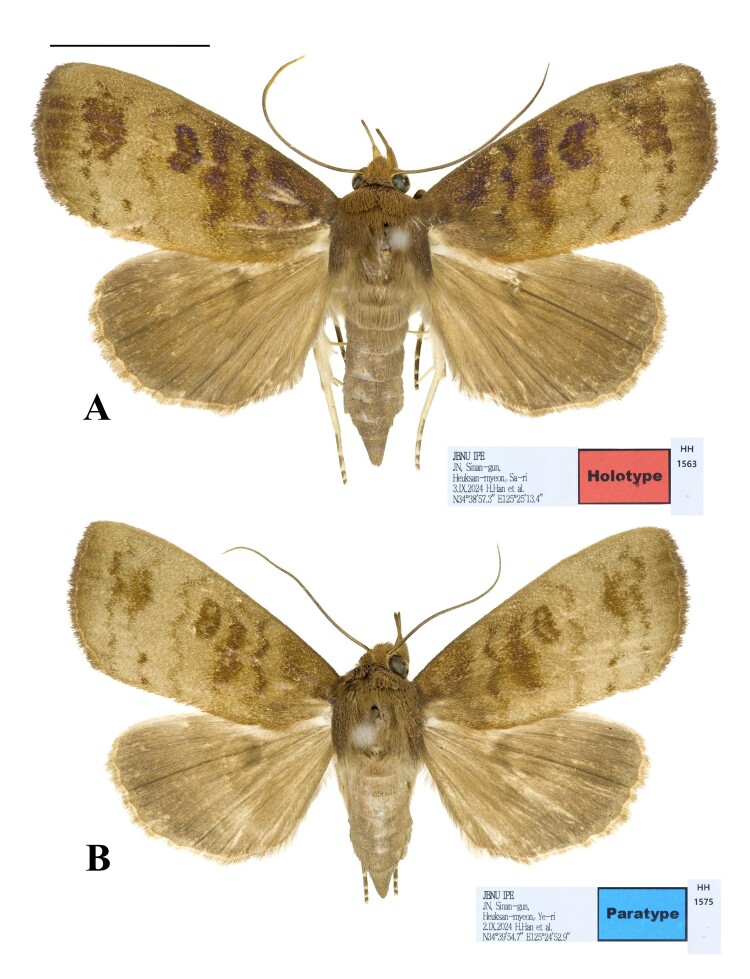
Type specimens of *Avitta fasciosa koreana* **ssp. nov. A** male; **B** female. Scale bar: 10 mm.

**Habitus**. (Figs 1, 5) Wingspan male 41 mm, female 36 mm. Ground colour golden brown. Eyes large and globular. Frons tuft, but clypeofrons naked. Antennae filiform. Proboscis well-developed. Labial palpus upturned; first segment short and tuft; second segment upward, thick, furry and as long as eye; third segment thin, weakly up-curved, as long as second one. Patagium brown, divided into two sections, hairy, not very long. Tegula brown, well-developed, covering whole thorax. Fore-wing obovate; basal field dull brown; basal line thick, purplish-brown, starting from costal, but halting at middle; antemedial field lighter than former field; antemedial line bilinear, proximal one thinner than basal line, undulate, shaded out at posterior margin, but distal band thick, fairly straight, but also fading out at posterior; medial field brighter than former, bearing kidney-shaped distinct reniform spot with slightly hollow middle; medial line very thick, both costal and posterior areas basally oblique, middle area irregularly, but fairly straight, touching reniform spot; postmedial field light; postmedial line also thick, crenate, weakly curved, partially faded between Cu1 vein and Cu2 vein; subterminal field same as former field, but with dark band bridging both postmedial and subterminal lines; subterminal line also thick, crenate, very weakly curved, irregularly and partially disappeared; terminal field proximally light, but distally dark brown; cilia short, dense, dull brown. Hind-wing uniformly weakly light brown; cilia dense, partially brighter than ground colour. Abdomen cornical, twice as long as thorax, uniformly greyish-brown.

**Figure 2.**
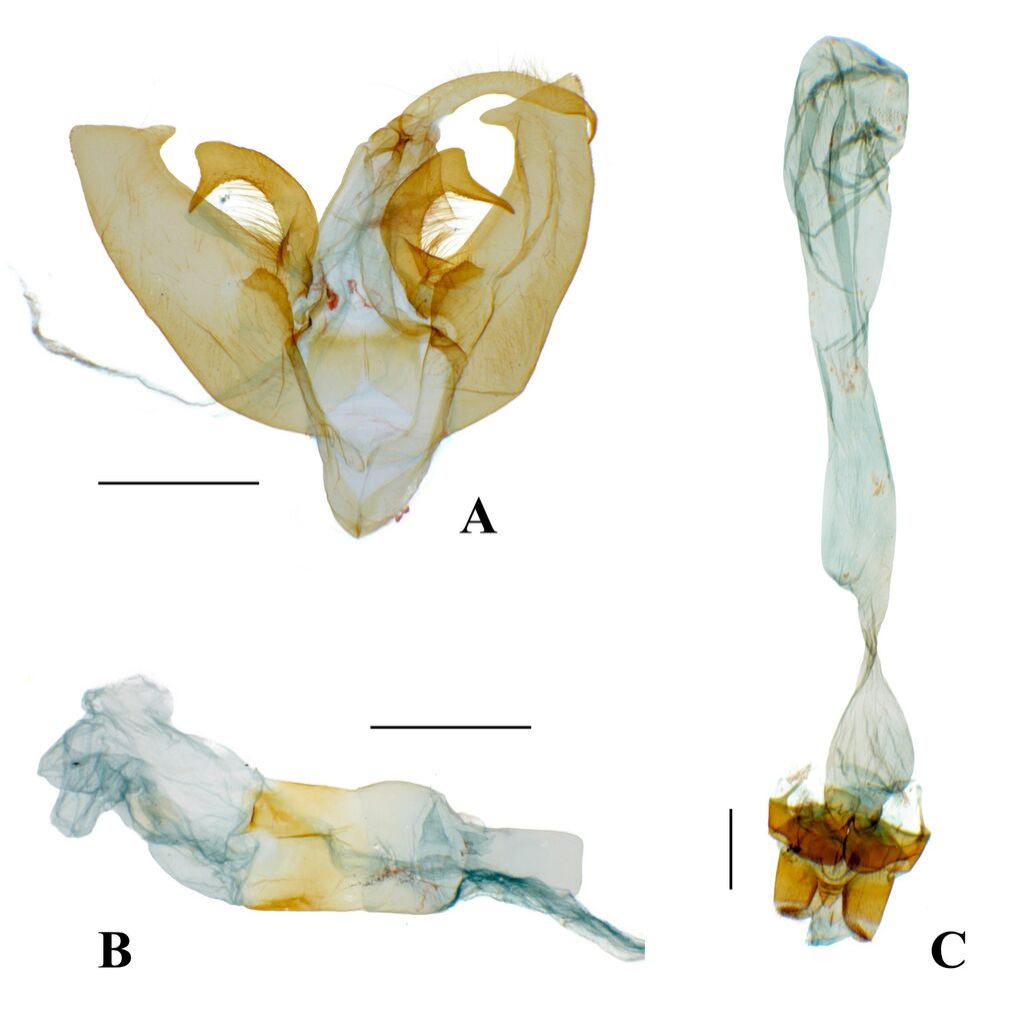
Genitalia of *A. f. koreana* **ssp. nov. A** male; **B** aedeagus; **C** female. Scale bar: 1 mm.

**Figure 3.**
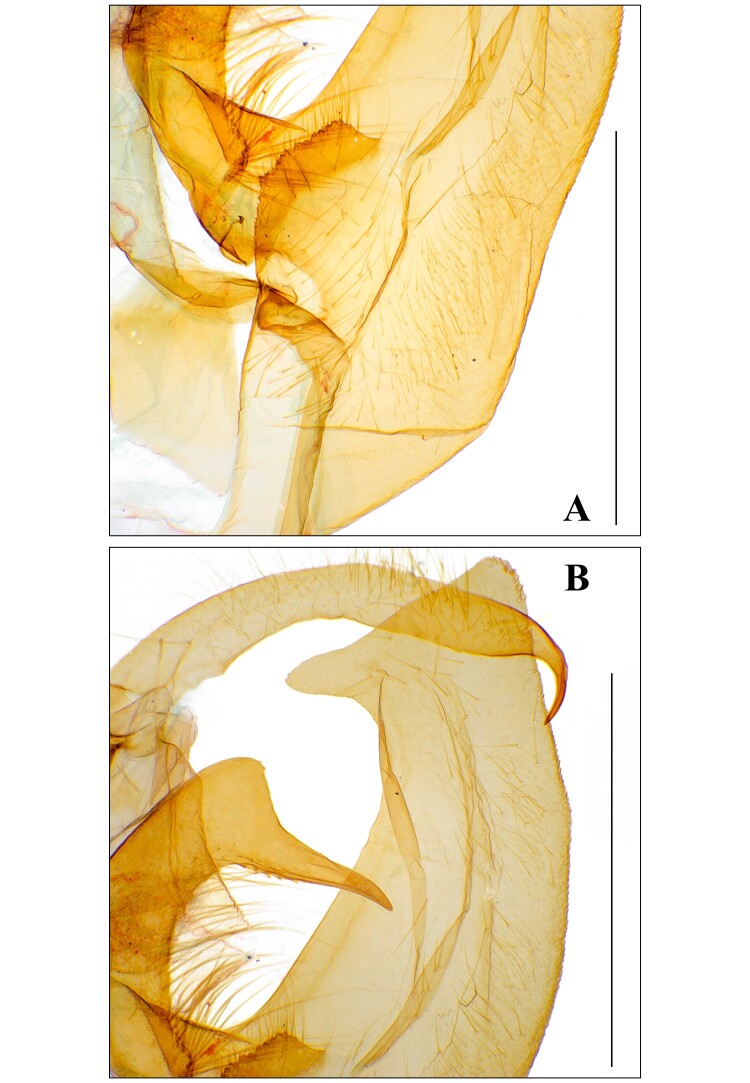
Male genitalia of *A. f. koreana* **ssp. nov. A.** basal area of left valva; **B.** costal process and top area of left valva. Scale bar: 1 mm.

**Figure 5.**
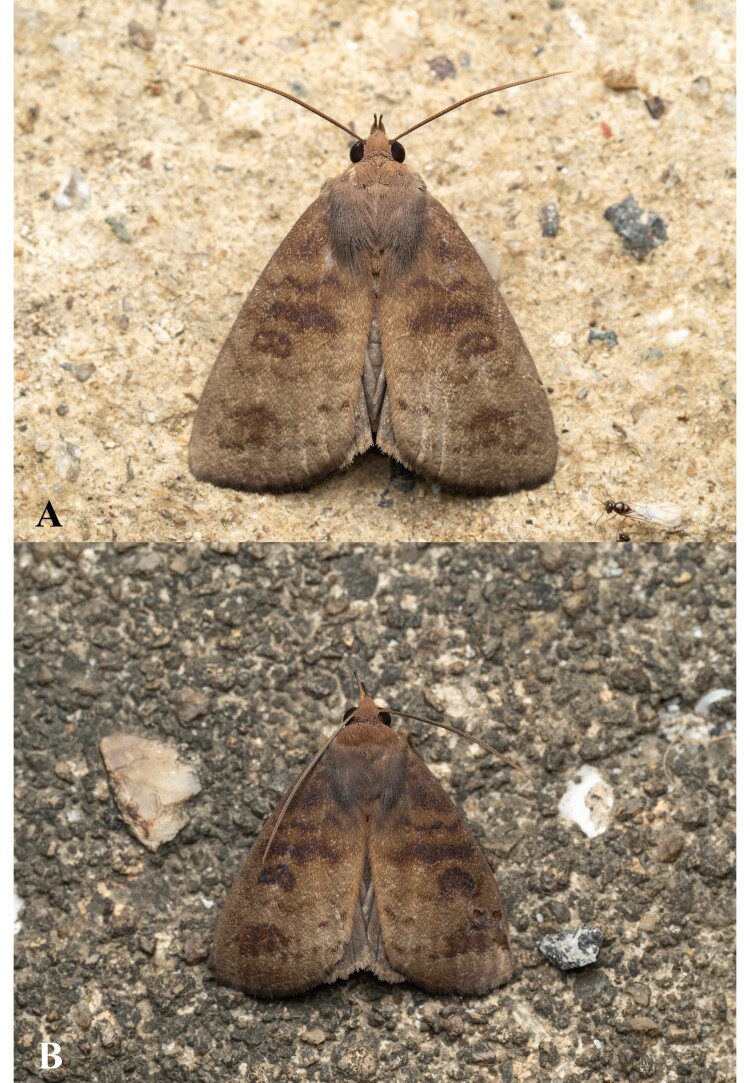
Photographs of living *A. f. koreana* **ssp. nov. A** male; **B** female. Taken by the first author.

**Male genitalia.** (Fig. 2A–C, Fig. 3) Fairly symmetrical. Uncus fairly curved, weakly setose, apically hooked strongly and thinner. Scaphium half as long as uncus, straight, weakly sclerotised. Tegumen straight, slightly longer than uncus. Vinculum really slightly shorter than tegumen, wholly curved slightly. Valva long and bearing distinct costal processes, but not tongue-like shape of typical *Avitta*; costal process asymmetrical, wholly curved, thick, bearing apical horn-like process, left one’s horn-like process fairly longer than right one’s; valva itself fairly thick, entirely curved, apically plate-like shape, bearing inwardly projected process; basal saccular process strongly sclerotised, rising near costal process; sacculus very slightly curved. Juxta slightly sclerotised, more sclerotised at top area, being divided and weakly sclerotised at bottom section. Aedeagus as long as tegumen, cylindrical, medially bulbous; coecum flat; ductus ejaculatorius rising from distal end of coecum; carina more sclerotised partially; vesica bulbous, bearing three short diverticula.

**Female genitalia.** (Fig. 2C) Papillae analis blunt, proximal three quarters sclerotised, but distal quarter(?) membranous, sparsely setose. Apophyses posteriors as long as anteriors, both two thirds as long as papillae analis. Lodix quite thin, strongly sclerotised, medially swollen. Ostium one third as long as papillae analis, opened, with sclerotised and inverted-deltoid outline. Antrum very short, sclerotised, cylindrical. Ductus bursae bulbous, four times as long as antrum, being very thin at meeting part with corpus bursae. Corpus bursae very long, four times as long as ductus bursae, elongate, becoming slightly thin at middle area, bearing an oblique, granulated signum band at top area.

#### Diagnosis

The species is very similar to *Avitta discipuncta* (Felder and Felder 1865) in their golden brown fore-wings, but they can be distinguished by the following characteristics: in habitus, fore-wing rectangular, narrower, with purplish-black fasciation which is more distinct in the basal of reniform (Holloway 2005). The new subspecies could be confused with *A. fasciosa fasciosa* and *A. fasciosa gracilis*, but can be distinguished by the following characteristics: in male genitalia, costal process thicker than *A. f. gracilis* (Fig. 2A, Fig. 3B and Fig. 4a) and its left apical horn-like process much longer than *A. f. fasciosa* (Fig. 2A, Fig. 3B and Fig. 4b); valva itself slender and curved (Fig. 2A and Fig. 3B), but *A. f. fasciosa* thick and fairly straight (Fig. 4b); apical area of valva flat (Fig. 2A and Fig. 3B), but *A. f. gracilis* rounded (Fig. 4a) and *A. fasciosa koreana* having inwardly projected process (Fig. 2A and Fig. 3B), but *A. f. fasciosa* with only a very short and small process (Fig. 4b); sclerotised area of basal valva distinct and situated at near the costa (Fig. 2A and Fig. 3A), but appearing near costa in *A. f. fasciosa* (Fig. 4b) and situated at middle part in *A. f. gracilis* (Fig. 4a).

#### Etymology

The subspecific epithet derived from the country where the specimen was collected (Korea, Heuksando Island); it is feminine in gender.

#### Distribution

Type locality: Heuksando Island, Korea.

#### Notes

*Avitta fasciosa gracilis* (Type Locality: Sarawak), a subspecies of the species *A. fasciosa* (Type Locality: Khasia Hills) from Borneo, is characterised by its shorter and thinner costal process and rounded valva tip, but Holloway (2005) treated it as only a subspecies of *A. fasciosa*. Having compared two types, one from northeast Himalaya and the other from Borneo, he concluded that their genital differences were insufficient to classify them as different species. *A. fasciosa koreana* **ssp. nov.** may be considered an eastern terminal form between the typical mainland Asian form and the Borneo-Sundaland form, though this is not assured. To resolve this, we suggest that the host plant of *A. fasciosa* should be further investigated. Although Hayashi (2003) reported *Cocculus trilobus* (Thunb.) DC. 1818 [Menispermaceae] as their host plant, given their separated distribution, more investigations are needed to study the precise relationships between the subspecies. Furthermore, there are quite distinct differences between their wingspans (Holloway (2005): 19–22 mm; this study: 36–41 mm). To check how considerable these differences are, it appears that more sampling is necessary. It is possible that these three subspecies could be elevated to species level or even downgraded, so further phylogenetic or population genetic studies using molecular data are needed to accurately evaluate their taxonomic levels.

## Identification Keys

### Key to subspecies of *Avitta fasciosa* Moore

**Table:** 

1	Costal process and valva thick; apical area of valva rounded and having very short and small inwardly projected process; sclerotised area of basal valva nearly appearing at costa	*A. f. fasciosa* Moore
–	Valva slender and curved	2
2	Costal process thin; apical area of valva just rounded; sclerotised area of basal valva intermediately situated	*A. f. gracilis* Holloway
–	Costal process thick; apical area of valva flat and having large inwardly projected process; sclerotised area of basal valva more distinct than congeners and situated near the costa	*A. f. koreana* **ssp. nov.**

## Supplementary Material

XML Treatment for Avitta

XML Treatment for Avitta fasciosa koreana

## Figures and Tables

**Figure 4a.**
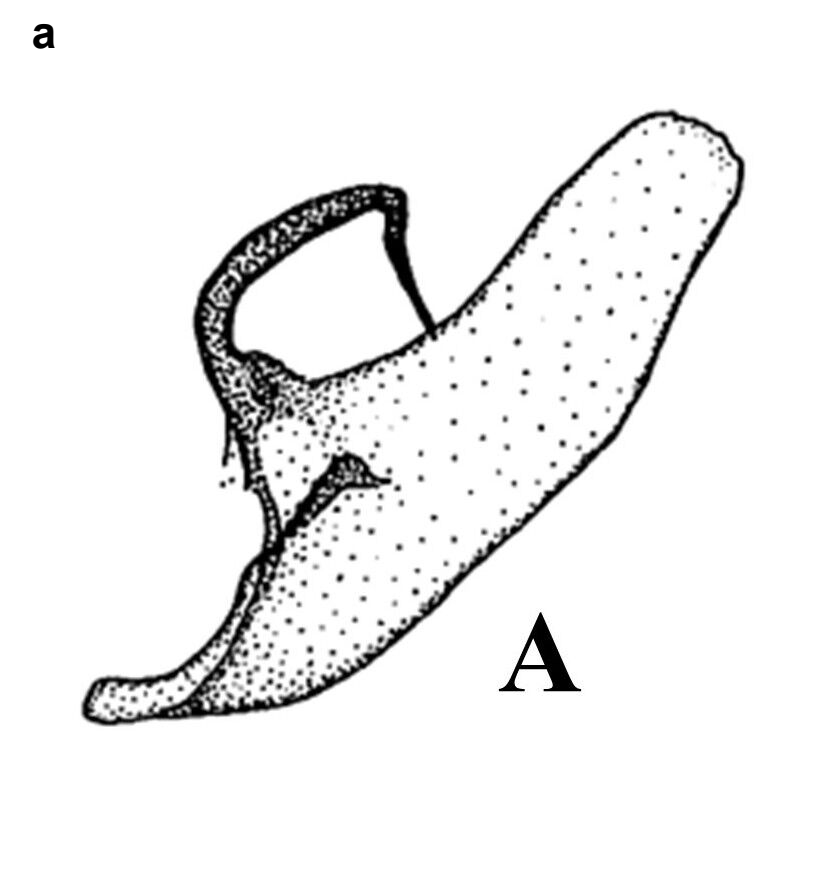
*A. f. gracilis*;

**Figure 4b.**
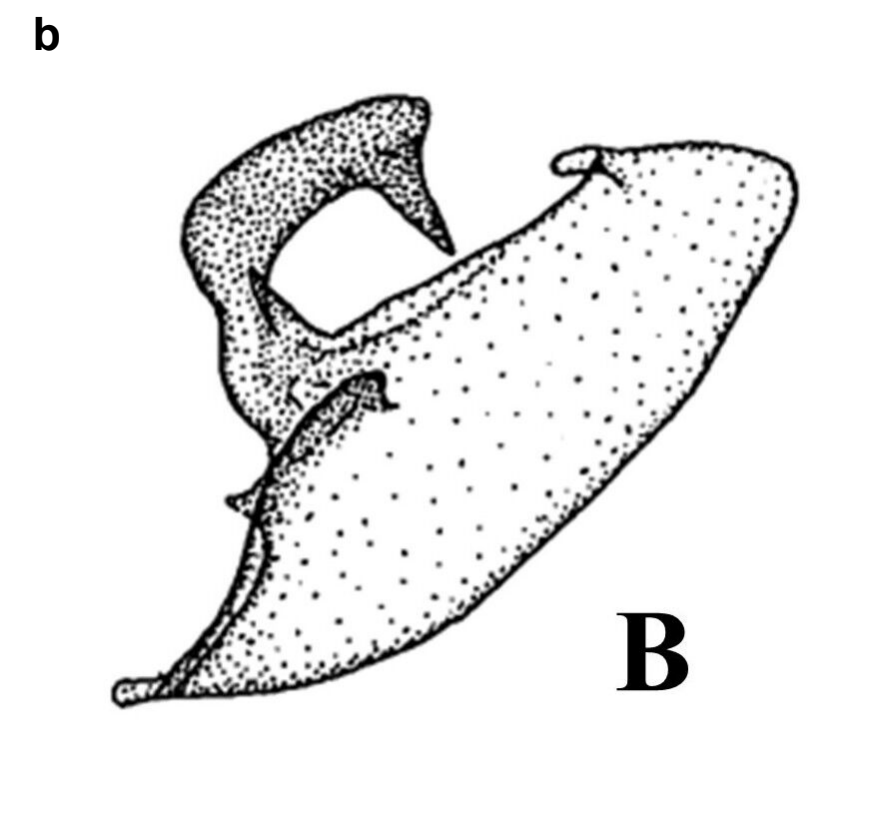
*A. f. fasciosa*.
